# Comparing the Performance Gap Between Males and Females in the Older Age Groups in IRONMAN® 70.3: An Internet-Based Cross-Sectional Study of More Than 800,000 Race Records

**DOI:** 10.1186/s40798-023-00636-x

**Published:** 2023-09-21

**Authors:** Beat Knechtle, David Valero, Elias Villiger, Mabliny Thuany, Marilia Santos Andrade, Pantelis T. Nikolaidis, Ivan Cuk, Katja Weiss

**Affiliations:** 1 Medbase St. Gallen am Vadianplatz, Vadianstrasse 26, 9001 St. Gallen, Switzerland; 2 Institute of Primary Care, University of Zurich, Zurich, Switzerland; 3 Ultra Sports Science Foundation, Pierre-Benite, France; 4 Klinik für Allgemeine Innere Medizin, Kantonsspital St. Gallen, St. Gallen, Switzerland; 5 Faculty of Sports, University of Porto, Porto, Portugal; 6 Department of Physiology, Federal University of Sao Paulo, São Paulo, Brazil; 7 School of Health and Caring Sciences, University of West Attica, Athens, Greece; 8 Faculty of Sport and Physical Education, University of Belgrade, Belgrade, Serbia

**Keywords:** Ultra-endurance, Swimming, Cycling, Running, Age group, Master athlete, Multi-sport, Sex differences

## Abstract

**Background:**

The sex difference in the three split disciplines (swimming, cycling, and running) and overall race times in triathlon races has mainly been investigated for the Olympic distance and IRONMAN® triathlon formats, but not for the half IRONMAN® distance, i.e., the IRONMAN® 70.3. The aim of the present study was to investigate the sex differences in IRONMAN® 70.3 by age group in 5-year intervals for the split disciplines of this race. Data from 823,459 records (625,393 males and 198,066 females) of all age group finishers (in 5-year intervals) competing in all official IRONMAN® 70.3 races held worldwide between 2004 and 2020 were analyzed, and sex differences by age group and split disciplines were evaluated.

**Results:**

Males were faster than females in all split disciplines and all age groups. The sex difference was lower in swimming than in cycling and running and less pronounced for triathletes between 20 and 50 years of age. After the age of 60 years, females were able to reduce the sex difference to males in swimming and cycling, but not in running, where the reduction in the sex difference started after the age of 70 years. The lowest sex difference was in the age group 75 + years for swimming and cycling and in the age group 30–34 years for running. Across age groups, the sex difference was U-shaped in swimming and running, with an increase after 18–24 years in swimming and after 40–44 years in running. In contrast, the sex difference decreased continuously with the increasing age for cycling.

**Conclusions:**

In conclusion, the study found that the sex difference in performance decreases with age in the IRONMAN® 70.3 race distance. However, females did not outperform males at older ages. Notably, sex differences were observed across different disciplines, with swimming displaying lower differences compared to cycling and running. These findings underscore the complex interplay between age, sex, and performance in endurance sports, emphasizing the need for additional research to understand the factors influencing these differences.

## Key Points

- Males outperformed females in all split disciplines, overall race times, and all age groups.
- Variations in sex differences were observed across different disciplines, with swimming displaying lower differences compared to cycling and running.
- Sex differences in performance decreased with age.

## Background

The sex difference in endurance performance is an important topic for athletes, coaches, and researchers in sports science and sports medicine. It seems to depend upon the discipline [1–3], the age of the athlete [3–5], the distance [6], and the duration of a performance [7–10]. Generally, males are faster than females in endurance disciplines [1, 11]. This has been reported for swimming [12–14], cycling [15, 16], and running [17, 18]. However, females were able to achieve a similar or even better performance than males in specific situations. Recent studies reported that females reduced the gap to males in swimming [7, 12, 19] and cycling [9, 20]. For long-distance swimming, it has been described that females can achieve similar performance to males [12, 21, 22]. Under certain circumstances, females in long-distance open-water swimming are even faster than males [23]. In distance-limited ultra-cycling races covering 100 miles, 200 miles, 400 miles, and 500 miles, males were faster than females in 100- and 200-mile races but not in 400- and 500-mile races [20]. In time-limited ultra-cycling races of 6 h, 12 h and 24 h duration, the sex differences in cycling speed decreased between males and females with increasing duration [9].

Reducing the sex difference in endurance performance also seems to depend on age. Several studies showed that reduced the gap between males with increasing age [7, 12, 19]. In swimming, the reduction of the sex difference has been shown in age group pool swimmers competing in all strokes such as backstroke [24], butterfly [25], breaststroke [19], freestyle [5], individual medley [26], and in 3000 m open-water swimming [12]. Regarding cycling, in both distance- (i.e., 100 miles, 200 miles, 400 miles, and 500 miles) [20] and time-limited (*i.e.*, 6 h, 12 h, and 24 h duration) ultra-cycling races [9], elderly females reduced the gap to elderly males. Considering running, females reduced the gap to males in the ultra-marathon running [27]. An analysis for different running distances and durations (*i.e.*, 5 km, 8 km, 10 km, 10 miles, 20 km, half-marathon, 25 km, 30 km, marathon, 50 km, 50 miles, 100 km, 100 miles, 12 h, 24 h, 48 h and 144 h) showed that the sex difference in performance decreased with increasing age but not with increasing distance or duration [28].

Triathlon is a multi-sports discipline consisting of swimming, cycling, and running where the long-distance triathlon races such as the ‘IRONMAN® Hawaii’ covering a 2.4-mile swim (3.9 km), a 112-mile bike (180.2 km), and a 26.2-mile run (42.2 km) are held officially since 1981 [29]. In recent years, however, the half-Ironman distance IRONMAN® 70.3 has become increasingly popular [30]. Each distance of the swim, bike and run segments is half the distance of that segment in a full IRONMAN® distance triathlon, where ‘70.3’ refers to the total distance in miles (113.0 km) of the race, consisting of a 1.2-mile (1.9 km) swim, a 56-mile (90 km) bike ride, and a 13.1-mile (21.1 km) run.

While sex differences are usually assessed in one sports discipline, the triathlon offers a model to study the variation of females’ and males’ performance across different locomotion patterns and environments (*i.e.*, swimming, cycling, and running) simultaneously. The aspect of sex difference in triathlon performance is well-investigated, especially for the Olympic distance triathlon covering a 0.93-mile (1.5-km) swim, a 24.8-mile (40-km) bike, and a 6.2-mile (10-km) run [31] and the IRONMAN®-distance triathlon [31]. The sex difference in the three split disciplines in a triathlon seems to depend upon the discipline [6, 32]. In both the Olympic distance triathlon [6] and the IRONMAN® distance triathlon [33], the sex difference in performance was less significant in cycling compared to swimming and running. Furthermore, the sex difference in performance depends upon age. In the age group triathletes, the sex difference in overall race time increased with increasing age [34]. While the sex difference increased after the age of 35 years in the Olympic distance triathlon [10], the sex difference increased after the age of 55–60 years in the IRONMAN® distance triathlon [4, 6]. In the IRONMAN® distance triathlon, the increase in the sex difference with increasing age depends upon the split discipline. In cycling and running, the sex difference in performance was greater in athletes of age groups older than 60 years than in athletes of younger ages [4].

While the aspect of sex difference in the split and overall race times has been well investigated in the Olympic distance triathlon [6, 33] and in the IRONMAN® distance triathlon [33, 35], to the best of our knowledge, no study has ever investigated the sex difference in the IRONMAN® 70.3 performance. With the lower barrier of entry, more athletes, including a potentially larger proportion of females, may be participating in IRONMAN® 70.3 races compared to the longer-distance IRONMAN® events. Therefore, the aim of the present study was to investigate the sex differences in the split disciplines (*i.e.*, swimming, cycling, and running) and overall race times by age group. Based upon the findings for both the Olympic distance triathlon and the IRONMAN® distance triathlon, we hypothesized to find performance differences between the split disciplines with a smaller sex difference in swimming compared to cycling. Considering the reduction of sex differences with age from the single disciplines where females’ participation rates are growing faster than the full IRONMAN® distance triathlon, we expected a reduction in the sex difference with increasing age where females would reduce the gap to males, especially in the higher age groups in IRONMAN® 70.3.

## Methods

### Ethical Approval

This study was approved by the Institutional Review Board of Kanton St. Gallen, Switzerland, with a waiver of the requirement for informed consent of the participants as the study involved the analysis of publicly available data (EKSG 01/06/2010). The study was conducted in accordance with recognized ethical standards according to the Declaration of Helsinki adopted in 1964 and revised in 2013.

### Data Set and Data Preparation

The race data was downloaded from the official IRONMAN® website (www.ironman.com) using a Python script (www.python.org) and consisted of over 1,3 million records of both professional (PRO) and amateur (age group or master) IRONMAN® 70.3 triathletes. Each record represents one triathlete´s registration in an IRONMAN® 70.3 competition and includes the athletes’ sex, age, country of origin, the year and the event location, the event status, and the times for swimming, running, cycling, and transitioning, among some other columns that were discarded. We analyzed all qualifying age group finishers of all IRONMAN® 70.3 races recorded on the official IRONMAN® website between 2004 and 2020. Data considered for this analysis were the times of each split discipline of the IRONMAN® 70.3 distance, considering swimming, cycling, running, and transition times (represented by transition 1—swimming for cycling, and transition 2—cycling for running) and the full race times (all in seconds), as well as the triathlete´s sex and age group. The athletes were sorted into 5-year age intervals (i.e., 18–24, 25–29, 30–34, 35–39, 40–44, 45–49, 50–54, 55–59, 60–64, 65–69, 70–74, 75–79, 80–84, and 85–89-years). Due to the low number of athletes older than 75 years, we combined the athletes from the age groups 75–79, 80–84, and 85–89 years in an overarching age group 75 + years. The exclusion criteria were (i) athletes who did not start or finish, (ii) disqualified athletes, (iii) records with missing split times, (iv) inconsistent times (i.e., impossible split times or final times smaller than split times, etc.) and (v) records missing essential information (i.e., age group, sex, etc.). Data were cleaned by discarding non-qualifying athletes (DNS = did not start, DNF = did not finish, DQ = disqualified) and excluding professional athletes. Furthermore, we removed duplicates, checked for and removed impossible data, excluded null data, harmonized age and sex data, normalized time formats, harmonized country and event data, renamed dataset columns as needed, and sex max/min filters to discard outliers. After processing the raw data, the resulting dataset consisted of 852,722 IRONMAN® 70.3 finishers’ records (645,639 males and 207,083 females), including amateur (age group or master) and professional triathletes’ records. Once professional triathletes records were excluded, the resulting dataset used in this analysis consisted of. 823,459 records (625,393 males and 198,066 females) IRONMAN® 70.3 age group finishers.

### Statistical Analysis

Histograms of the split and full race times by sex were plotted for the full sample of age group triathletes, showing a normal distribution in all cases except for the run time, which is a bit right skewed. Violin plots were used to compare the split and full race times between the age groups and sexes, with statistical significance tested through ANOVA two-way tests. The change percentage between male and female average times in each group was also calculated for each split discipline and overall race time. Descriptive statistics are presented using mean, standard deviation, frequencies, and percentages. All analysis was done in a Colab Notebook (https://colab.research.google.com/) with Python (www.python.org/) and associated libraries.

## Results

**Fig. 1:**
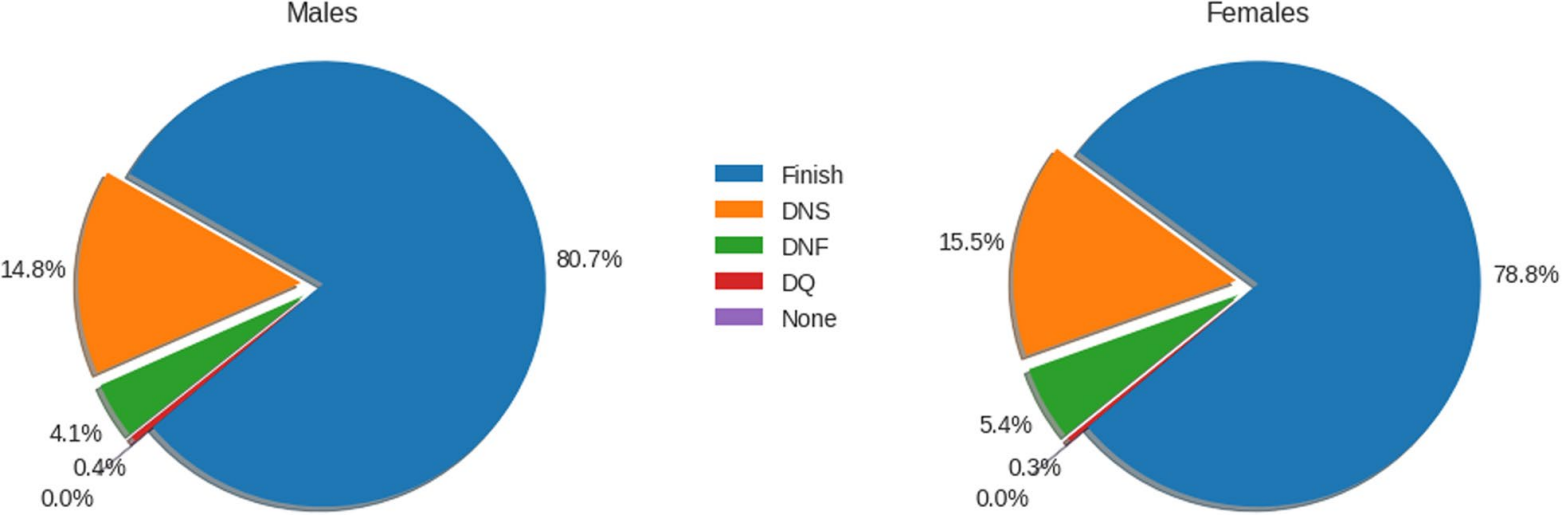
Participants by event status and sex (DNS = Did Not Start; DNF = Did Not Finish; DQ = Disqualified)

Figure 1 shows in a pie chart the percentage of triathletes of each sex by event status. Around 20% of all participants who registered did not finish the race.

**Fig. 2:**
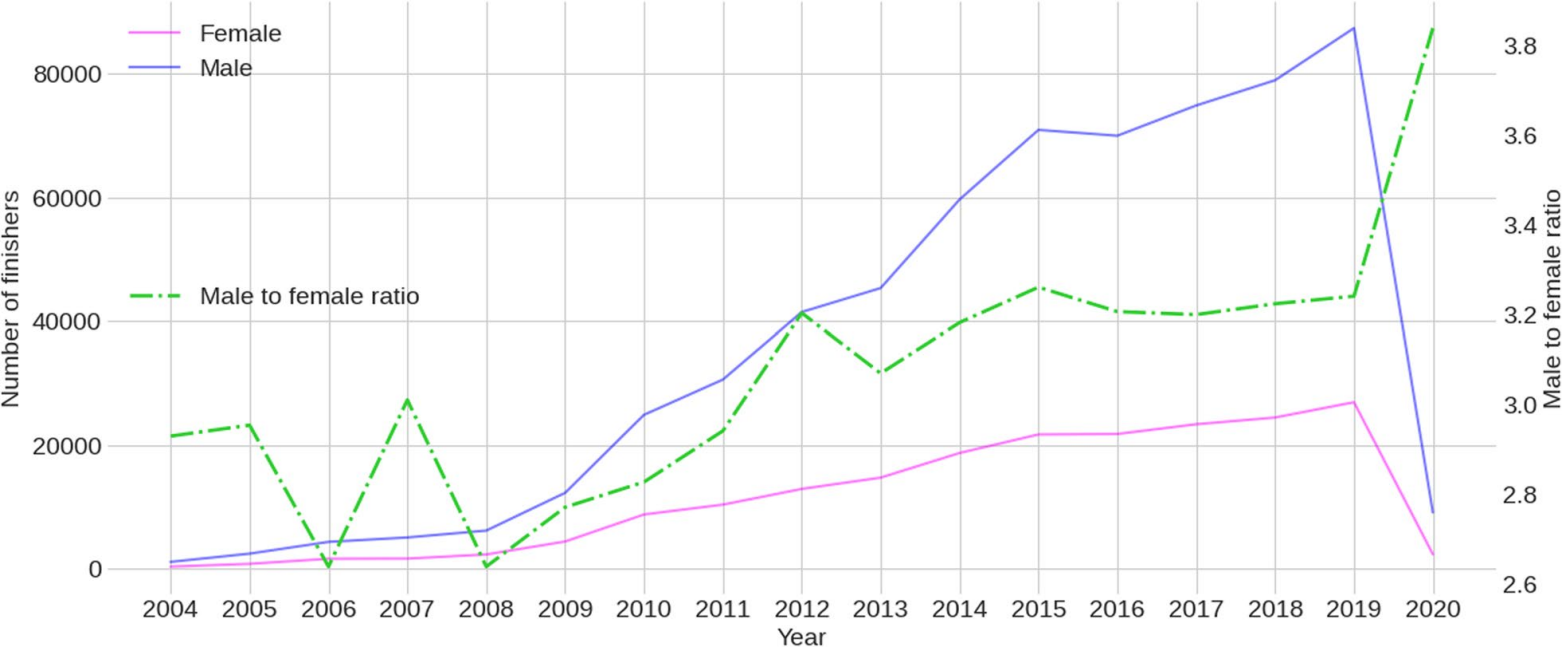
The female and male finishers trend between 2004 and 2020

Figure 2 shows the female and male finishers trend between 2004 and 2020. The number of male finishers increased more than the number of female finishers. In 2020, the number of finishers was dramatically reduced due to the COVID pandemic.

**Fig. 3:**
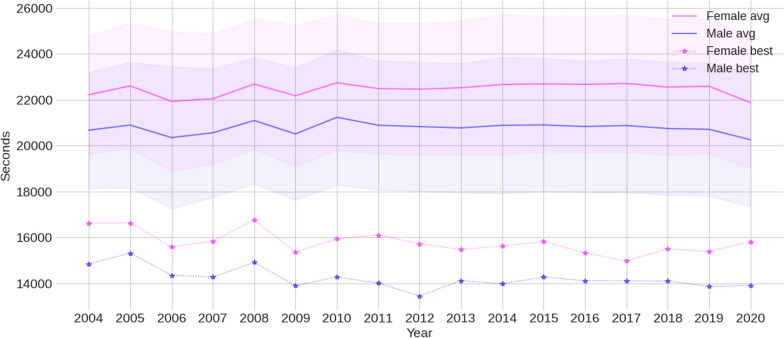
The average (solid line) ± standard deviation (colored bars) and best race times for male and female participants in the Ironman 70.3 between 2004 and 2020

Figure 3 presents the trend of the average and best race times between 2004 and 2020. From 2010 it is possible to verify performance stability in both sexes, with a decline between 2019 and 2020.

Table 1 summarizes the number of finishers per age group and the corresponding statistical values of the race times. For females, most finishers were in the age group 35–39 years, whereas the fastest females were in the age group 25–29 years. For males, again, most finishers were in the age group 35–39 years, but the fastest males were in the age group 18–24 years. Overall, race times increased with increasing age.

**Table 1:** Descriptive information per age group in both sexes

Age group	Overall finish times hh:mm:ss (Females)					Overall finish times hh:mm:ss (Males)					Males-to-females ratio
	n	Mean	Std	Min	Max	n	Mean	Std	Min	Max	
18–24	8129	06:10:20	00:49:42	04:16:35	09:20:33	22,484	05:38:38	00:51:04	03:51:35	09:25:05	2.76
25–29	26,130	06:09:29	00:49:16	04:09:34	09:42:12	63,093	05:40:45	00:50:00	03:51:31	09:13:07	2.41
30–34	35,555	06:10:04	00:49:17	04:18:56	09:38:53	102,127	05:41:16	00:48:39	03:44:09	09:32:12	2.87
35–39	37,189	06:13:18	00:49:06	04:17:54	09:58:19	123,729	05:43:27	00:47:38	03:52:49	09:41:22	3.32
40–44	35,910	06:16:18	00:48:30	04:16:58	09:41:35	123,659	05:46:34	00:46:44	03:53:09	09:30:51	3.44
45–49	26,993	06:20:14	00:47:10	04:16:24	09:42:21	92,523	05:49:47	00:46:15	03:59:34	10:08:49	3.42
50–54	16,879	06:26:25	00:47:17	04:29:09	09:41:47	55,880	05:54:25	00:45:53	04:10:00	09:20:56	3.31
55–59	7664	06:35:04	00:46:55	04:46:30	09:30:18	26,448	06:01:54	00:45:31	04:06:51	09:27:47	3.45
60–64	2766	06:49:32	00:47:40	04:52:12	09:45:20	10,332	06:13:00	00:46:09	04:23:09	09:39:09	3.73
65–69	665	07:05:10	00:42:24	05:10:06	09:18:45	3730	06:28:14	00:45:47	04:45:33	09:07:59	5.60
70–74	165	07:28:41	00:44:56	06:01:25	09:25:09	1142	06:52:49	00:44:10	05:06:02	09:28:55	6.92
75 +	21	07:43:26	00:46:32	06:21:01	09:13:50	246	07:18:36	00:46:55	04:23:22	08:57:47	11.71

n (sample size); std (standard deviation); min (minimum values); max (maximum values)

**Fig. 4:**
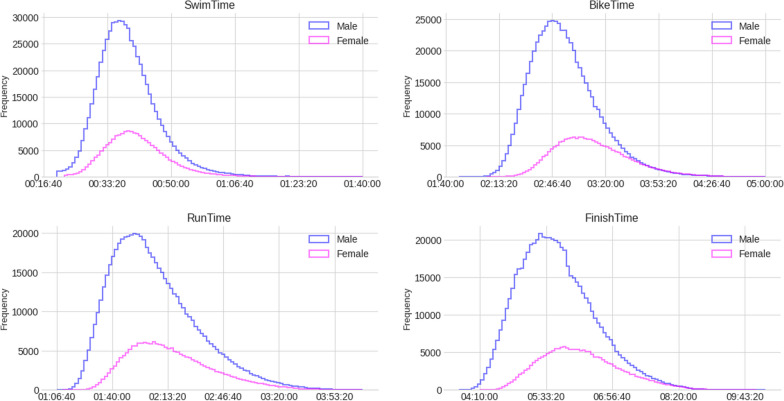
Split and overall race times histograms for females and males

Figure 4 shows the split and overall race times histograms for females and males. For females, the mean swim times were 40:55min:s (SD 08:08min:s), the mean cycling times 03:11:34h:min:s (SD 23:06h:min), the mean running times 02:15:01h:min:s (SD 25:22min:s), and the mean overall race times 06:16:18h:min:s (SD 51:25min:s). For males, the mean swim times were 38:42min:s (SD 07:52min:s), the mean cycling times 02:54:29h:min:s (SD 21:18min:s), the mean running times 02:05:14h:min:s (SD 26:10min:s), and the mean overall race times 05:46:53h:min:s (SD 48:10min:s).

**Fig. 5:**
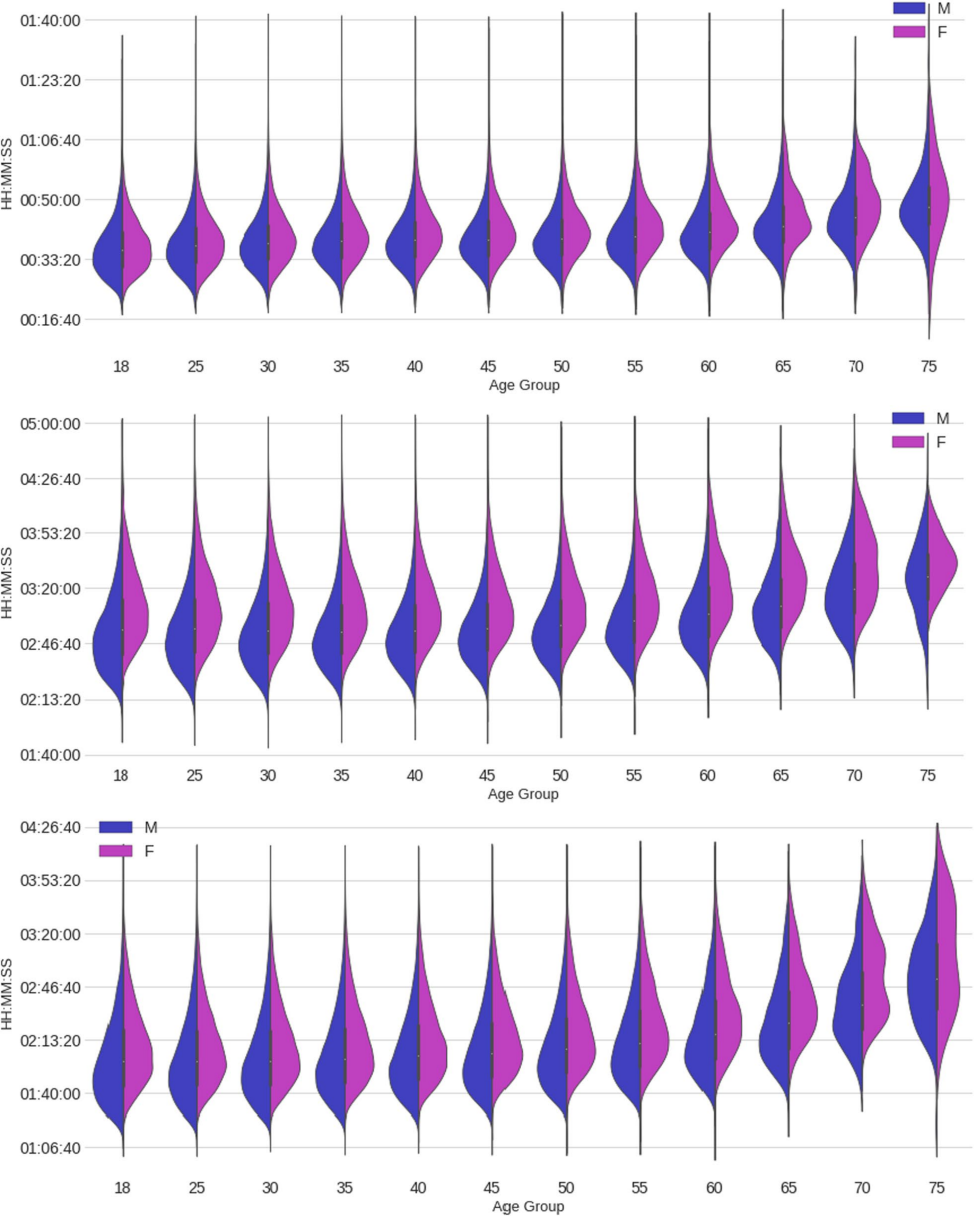

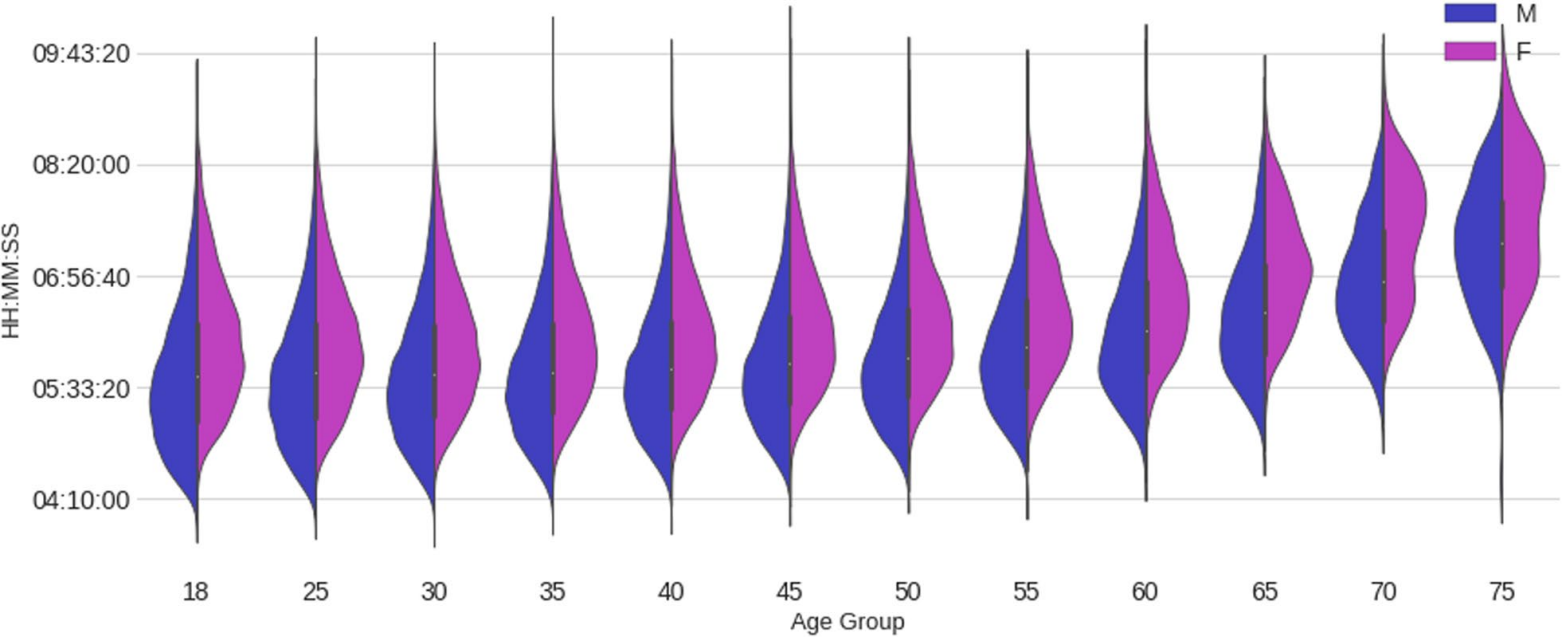
Violin plots of split and full race times by age group and sex. Swimming, cycling, running and overall race time from top to bottom

Figure 5 presents a set of violin plots showing the split and overall race time distribution by age group and sex. For all split disciplines and overall race times, males were always faster than females in all groups, although the difference is smaller in the swimming discipline than in the other two. It is worth noticing how close male and female swim time boxes are, with significant overlap, mainly in the low and mid-age groups compared to the bike or run splits (or to the overall finish time). This suggests that the performance of males and females in the water (swimming) is less different between the sexes than on land (running and cycling). Also worth noticing is how the cycling times remain flatter than running and swimming times through the lower and middle age groups. This suggests that the cycling discipline exhibits a more consistent performance over the years.

ANOVA two-way tests were applied to the split and overall race times (Table 2). The results showed that, for each independent variable (i.e., age group and sex) and their interaction, the calculated *p* values PR(> F) were zero or almost zero, and hence it can be concluded that the differences between age groups and sexes are statistically significant. The ANOVA test results tables follow:

**Table 2:** ANOVA test results to the split and overall race time

Finish time	sum_sq	Df	F	*p* value
Intercept	4.01e+12	1	488,589	< 0.001
AgeGroup	4.94e+10	11	547	< 0.001
Sex	2.16e+10	1	2627	< 0.001
AgeGroup:Sex	1.18e+09	11	13	< 0.001
Residual	6.76e+12	823,435	NaN	NaN
*Swim time*				
Intercept	4.17e+10	1	186,850	< 0.001
AgeGroup	1.32e+09	11	536	< 0.001
Sex	3.73e++07	1	167	< 0.001
AgeGroup:Sex	1.25e+08	11	51	< 0.001
Residual	1.84e+11	823,435	NaN	NaN
*Bike time*				
Intercept	1.09e+12	1	646,399	< 0.001
AgeGroup	3.62e+09	11	195	< 0.001
Sex	7.76e+09	1	4619	< 0.001
AgeGroup:Sex	1.66e+08	11	9	< 0.001
Residual	1.38e+12	823,435	NaN	NaN
*Run time*				
Intercept	5.10e+11	1	220,121	< 0.001
C(AgeBand)	1.66e+10	11	650	< 0.001
C(Sex)	2.61e+09	1	1128	3.44e− < 0.001
C(AgeBand):C(Sex)	7.72e+08	11	30	< 0.001
Residual	1.91e+12	823,435	NaN	NaN

'FinishTime ~ C(AgeBand) + C(Sex) + C(AgeBand):C(Sex)';'SwimTime ~ C(AgeBand) + C(Sex) + C(AgeBand):C(Sex)'; 'BikeTime ~ C(AgeBand) + C(Sex) + C(AgeBand):C(Sex)'; 'RunTime ~ C(AgeBand) + C(Sex) + C(AgeBand):C(Sex)'

**Fig. 6:**
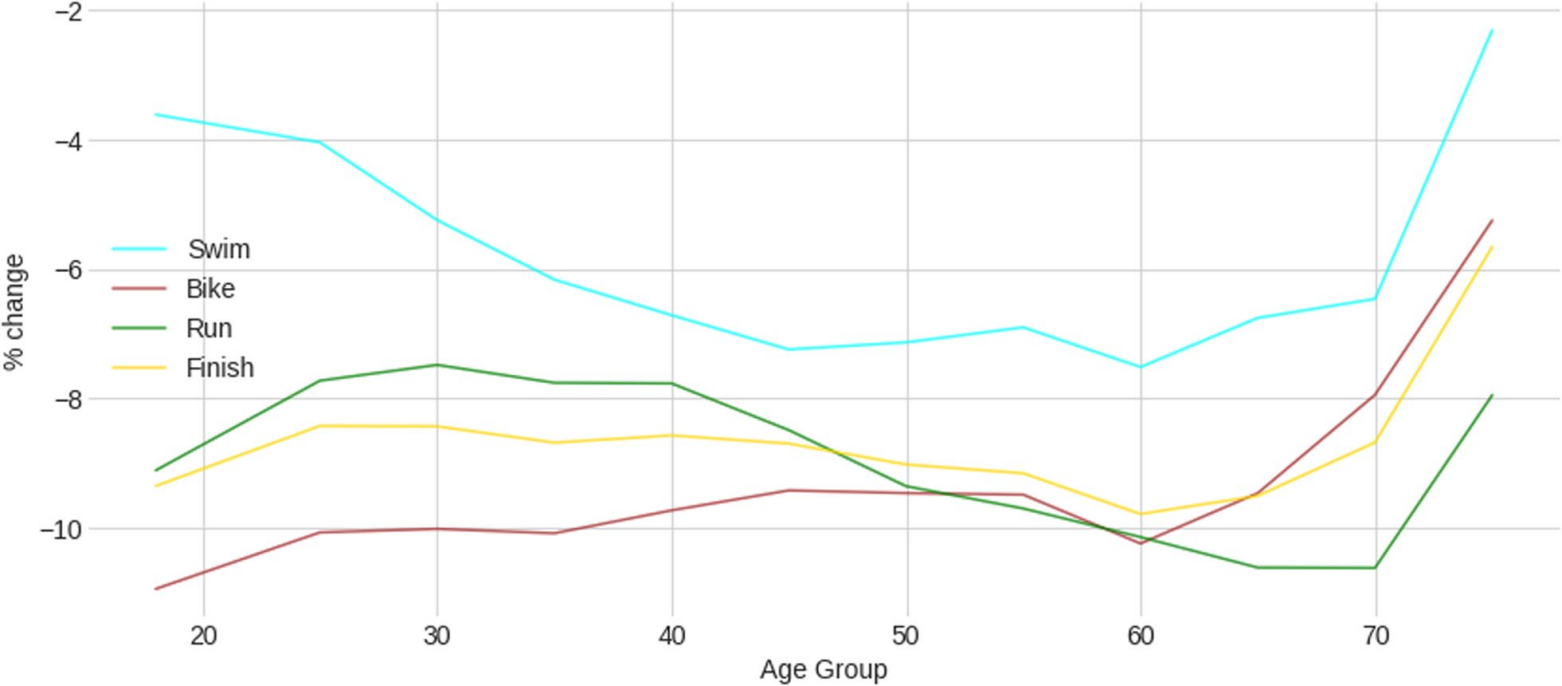
Percent change in time between females and males by age group and split disciplines

Figure 6 presents the percent difference in time between females and males by age group and split disciplines. Males are always faster than females in all age groups. The sex difference was lower in swimming compared to running and cycling. In swimming, the lowest sex difference was in the age group 18–24 years (3.66%). In cycling, the lowest sex difference was in the age group 70–74 years (4.13%). In running, the lowest sex difference was in the age group 30–34 years (7.46%). The differences were less pronounced in swimming than in cycling and running between 20–24 and 50–54 years. After the age of 60–64 years, females were able to reduce the sex difference to males in swimming and cycling, but not in running, where the reduction started in the age group 70–74 years. The sex difference showed a kind of U-shaped curve in swimming and running, with an increase after 18–24 years in swimming and after 40–44 years in running. In contrast, for cycling, the sex difference decreased continuously with increasing age.

## Discussion

This study intended to investigate the sex difference in performance with increasing age in the IRONMAN® 70.3 triathletes by split discipline and overall race times, with the hypotheses that differences would exist between the split disciplines, with a smaller difference in swimming performance compared to cycling, and a reduction in the difference with age, where older females would be able to reduce the gap to males. Males were always faster than females. The sex differences in swimming were less pronounced than in cycling and running for all age groups. The differences were smaller in swimming for all age groups. After the age of 60 years, females were able to reduce the difference with males in swimming and cycling, but not in running, where the reduction in difference started after the age of 70 years. The lowest sex difference was observed in the age group 75 + years for both swimming and cycling and in the age group 30–34 years for running. Across all age groups, the sex difference was U-shaped for swimming and running, with an increase after 18–24 years in swimming and after 40–44 years in running. For cycling, the sex difference decreased continuously with increasing age.

### Males Were Faster Than Females

The first important finding was that males were faster than females in all split disciplines, overall race times, and all age groups. The better performance of males compared to females is most likely due to biological differences. Generally, male triathletes tend to have a higher muscle mass and bone density, potentially providing them with advantages in strength and power. On the other hand, female triathletes may possess a higher percentage of body fat, which can contribute to better buoyancy in the water. Hormonal variations also play a role, as males typically have higher testosterone levels, potentially enhancing their muscle development and aerobic capacity. However, it's crucial to recognize that individual variations exist, and training, nutrition, and genetics significantly impact athletic performance. Moreover, age is another factor in triathlon. Younger triathletes often possess greater speed, agility, and recovery capacity, enabling them to excel in short distances. In contrast, older triathletes may have accumulated experience, mental fortitude, and endurance, making them well-suited for longer distances where pacing and endurance play a crucial role [17, 36, 37].

We need to consider in this context the age-related performance decline. Overall, endurance performance depends upon age [3, 38] and declines with increasing age [11, 39, 40]. An age-related performance decline is well-described [13, 32, 41] and accounts for a ~ 1.25% decline per year [42]. The start of the age-related decline in performance seems to depend upon the sports discipline [32, 35], the kind of physical activity [38], and the sex [3, 32]. Generally, the age-related performance decline starts at ~ 30–35 years [32], remains fairly stable between ~ 35 and ~ 50 years [3, 38], decreases linearly until the age of ~ 70 years [43], and starts to decrease exponentially after the age of ~ 70 years In the IRONMAN® 70.3, the oldest age group was 75–79 years and no athletes older than 80 years were recorded (see Table 1). Therefore, we cannot compare the sex differences in the 80 + age group.

Another consideration is the decrease in the sex difference in the 70 + age group (Fig. 6). This might be because only a very selected group of high-performing females compete at these old ages. The sample of males also includes older ages, bringing the average down and helping to shorten the gap between males and females.

### Smaller Sex Difference in Swimming Compared to Cycling and Running

A second important finding was that the sex difference in performance was smallest in swimming. For cycling and running, the difference in cycling was higher in younger and lower in older age groups, whereas for running, the difference was lower in younger and higher in older age groups. Our hypothesis to find a smaller sex difference in performance in swimming compared to cycling could be confirmed.

Generally, sex differences in endurance performance seemed to depend upon the discipline. It has been reported that sex differences were generally smaller in swimming compared to running [2, 3, 35] and cycling [3, 16, 35]. A very likely explanation that females reduced the gap to males in swimming can be attributed to their distinct body shape, characterized by higher body fat, which provides enhanced insulation against the cold water and improved buoyancy in the leg region and differs from that of male swimmers who tend to accumulate fat in the central region [45]. During dynamic swimming with varying velocities, the swimmer generates movement in the surrounding water, akin to an additional mass. Female swimmers exhibit a lower absolute and relative added mass compared to male swimmers, indicating that differences in body shape between the sexes may be associated with this added mass phenomenon [46].

In the Olympic distance triathlon and the Ironman distance triathlon, the sex difference was lower in cycling compared to swimming and running [6, 33]. We found sex differences for cycling and running across all age groups. Considering the age-related performance decline again, the decline differs in triathlon between the different race distances [34, 47] and the split disciplines [47]. Regarding the split disciplines, the age-related performance decline in triathlon is less pronounced in cycling compared to swimming and running [32, 34]. Regarding the race distances, the magnitude of the performance declines in cycling and running performances with advancing age for the Olympic distance triathlons are less pronounced than for the Ironman distance races [34] and the age-related performance decline in cycling and running is greater in the Ironman triathlon than in the Olympic distance triathlon [47]. The differences in cycling and running across age groups in Ironman® 70.3 athletes might be explained by the difference in split distances between the Olympic and the Ironman distance triathlon.

### Sex Differences by Split Disciplines and Age Groups

A third important finding was that females were able to reduce the sex difference to males after the age of 60 years in swimming and cycling, but not in running where the reduction in sex difference started later at the age of 70 years. Since the number of finishers decreased with increasing age, this might lead to a selection bias and may reduce the statistical significance. However, an increase in participation of age group athletes in recent decades has been reported for different sports disciplines such as marathon running, ultra-marathon running [48], and long-distance triathlon [47]. It has been shown that master or age group athletes have improved performance in different sports disciplines such as swimming [19], ultra-marathon running [49], and Olympic distance triathlon [6].

Age group athletes seem to be able to dominate certain sports disciplines such as ultra-marathon running [49]. Several studies showed that the best athletes in endurance performance became faster as they got older. This has been reported in the ultra-cycling [26], long-distance open-water swimming [23] and long-distance triathlon [50]. Furthermore, the number of female age group athletes has increased more than the number of male age group athletes in sports disciplines such as marathon [48] and triathlon [34]. These aspects might explain that the reduction in sex difference started later in age in running compared to swimming and cycling.

A further interesting finding was that the split disciplines showed differences regarding sex difference. The least significant sex differences were in the age group 75 + years in swimming and cycling and in the age group 30–34 years in running. The differences in these three split disciplines might be explained by the physical, physiological, and anthropometric differences between females and males [1, 51–53]. However, females have lower muscular fatigability and faster recovery during endurance exercise [53]. The lower fatigability might be due to the greater proportional area of type I muscle fibers [51]. Furthermore, swimming is a non-weight-bearing sports discipline, such as is cycling, whereas running is a weight-bearing discipline.

A last important finding regarding the sex differences was the different curves for the three split disciplines. In swimming, the sex difference followed a U-shaped curve with a less significant sex difference in the age group 18–24, increased until the age group 40–74 and decreased in the age group 70–74 years. In other words, female IRONMAN® 70.3 triathletes seemed to be able to reduce the gap to males after the age of 75 + years in swimming. It has been well-described that elderly females were able to reduce the gap to elderly males in pool swimming, where females achieved a similar performance after the age of 80 years and older for different strokes and disciplines [7, 19, 25]. The swim distance seems, however, to be relevant. While pool swimmers compete in no longer than 1500 m freestyle [54], the IRONMAN® 70.3 triathletes must swim 1.9 km during their competition. Female swimmers can achieve a similar performance for longer swimming distances in the age group 75–79 as male swimmers in 3000 m open-water swimming [12].

A limitation of the present study was the specific characteristics of this triathlon format [55, 56]. For instance, it has been observed that the IRONMAN® 70.3 had different predictors than Sprint- and Olympic-distance triathlon and the IRONMAN® 140.6 [56]. Thus, caution would be needed when generalizing our findings to other triathlon formats. Further limitations to consider are the wide age range of participants from different socio-cultural backgrounds which complicates the understanding of changes in sex differences, and the lower number of females in each age group influencing the results as seen in running. The strength of our study was the large dataset that was analyzed, which allowed drawing safe conclusions about the variation of sex differences by age and discipline in the IRONMAN® 70.3. These findings have practical applications for triathletes, especially in setting long-term goals, and could help coaches optimize their training routine in the context of their age and sex accordingly and achieve better final results.

## Conclusions

In conclusion, the findings suggest that, for the IRONMAN® 70.3 race distance, the sex difference between males and females becomes less pronounced with increasing age, similar to what has been observed in single endurance disciplines such as swimming. However, females were not able to outperform or achieve parity with males at older ages, as has been reported in other individual distances like swimming and cycling. The patterns of sex differences across the split disciplines did not follow a consistent trend and were mostly U-shaped across all age groups. Notably, swimming exhibited lower sex differences compared to cycling and running across all age groups. Overall, these results emphasize the complex interplay between age, sex, and performance in endurance sports. While females demonstrated a reduction in the sex difference with age, their performance did not surpass that of males in later years. These findings underscore the need for further exploration of factors influencing sex differences in endurance events and the broader context of performance in athletic competitions.

## Data Availability

For this study, we have included official results and split times from the official IRONMAN® website (www.ironman.com).

## Abbreviations

ANOVA: Analysis of variance
DNS: Did not start
DNF: Did not finish
DQ: Disqualified
Pro: Professional
